# PPARγ Agonists Attenuate Palmitate-Induced ER Stress through Up-Regulation of SCD-1 in Macrophages

**DOI:** 10.1371/journal.pone.0128546

**Published:** 2015-06-10

**Authors:** Jiro Ikeda, Toshihiro Ichiki, Yusuke Takahara, Hiroshi Kojima, Chikahiro Sankoda, Shiro Kitamoto, Tomotake Tokunou, Kenji Sunagawa

**Affiliations:** 1 Departments of Cardiovascular Medicine, Kyushu University Graduate School of Medical Sciences, Fukuoka, Japan; 2 Advanced Therapeutics for Cardiovascular Diseases, Kyushu University Graduate School of Medical Sciences, Fukuoka, Japan; University of Hong Kong, HONG KONG

## Abstract

**Background:**

Clinical trials have shown that treatment of patients with type 2 diabetes with pioglitazone, a peroxisome proliferator-activated receptor (PPAR)γ agonist, reduces cardiovascular events. However, the effect of PPARγ agonists on endoplasmic reticulum (ER) stress that plays an important role in the progression of atherosclerosis has not been determined. We sought to determine the effect of PPARγ agonists on ER stress induced by palmitate, the most abundant saturated fatty acid in the serum.

**Methods and Results:**

Protein expression of ER stress marker was evaluated by Western blot analysis and stearoyl-CoA desaturase1 (SCD-1) mRNA expression was evaluated by qRT-PCR. Macrophage apoptosis was detected by flowcytometry. Pioglitazone and rosiglitazone reduced palmitate-induced phosphorylation of PERK, a marker of ER stress, in RAW264.7, a murine macrophage cell line. Pioglitazone also suppressed palmitate-induced apoptosis in association with inhibition of CHOP expression, JNK phosphorylation and cleavage of caspase-3. These effects of pioglitazone were reversed by GW9662, a PPARγ antagonist, indicating that PPARγ is involved in this process. PPARγ agonists increased expression of SCD-1 that introduces a double bond on the acyl chain of long-chain fatty acid. 4-(2-Chlorophenoxy)-N-(3-(3-methylcarbamoyl)phenyl)piperidine-1-carboxamide, an inhibitor of SCD-1, abolished the anti-ER stress and anti-apoptotic effects of pioglitazone. These results suggest that PPARγ agonists attenuate palmitate-induced ER stress and apoptosis through SCD-1 induction. Up-regulation of SCD-1 may contribute to the reduction of cardiovascular events by treatment with PPARγ agonists.

## Introduction

The endoplasmic reticulum (ER) is an important organelle required for cell survival and normal cellular function, such as regulation of protein synthesis, appropriate folding of protein, and calcium storage. Various insults including ischemia, hypoxia and a high-fat diet induce accumulation of unfolded proteins in ER, which is designated ER stress [1]. In response to ER stress, the unfolded protein response (UPR) occurs to avoid apoptosis. The UPR consists of three main signaling systems initiated by three ER localized stress sensors such as PERK, IRE1 and ATF6. These molecular transduction systems attempt to maintain protein folding capacity and prevent a buildup of unproductive or toxic misfolded proteins. However, severe or prolonged ER stress can trigger apoptosis, which is promoted by transcriptional induction of CHOP/GADD153, activation of c-JUN N-terminal kinase (JNK), and caspase-dependent pathways [2].

Atherosclerosis is a chronic inflammatory disease and the leading cause of death in developed countries. ER stress and UPR activation are markedly increased in all stages of atherosclerotic lesions in an animal model and human atherosclerotic lesion [3, 4]. Macrophage apoptosis also occurs various stages of atherosclerosis. Apoptosis of macrophage occurs in macrophage-rich regions and cleared safely in early atherosclerotic lesions, which is associated with smaller lesion size [5, 6]. However, recent studies suggest that macrophage apoptosis induced by severe or prolonged ER stress is one of the critical mechanisms that induce vulnerability of the atherosclerosis plaque [7]. It has been reported that Fabp4 deficiency-induced LXRα up-regulation in macrophage resulted in an increase in the activity of stearoyl CoA desaturase-1 (SCD-1), an enzyme that converts endogenous saturated fatty acids (SFA) to monounsaturated fatty acids (MUFA), and thereby reduced ER stress and atherosclerosis [8]. Because peroxisome proliferator-activated receptor (PPAR) γ agonists have been reported to increase expression of LXRα and ABCA1 and attenuate atherosclerosis [9], we hypothesized that thiazolidinediones (TZD), synthetic PPARγ agonists, may attenuate ER stress induced by SFA through up-regulation of SCD-1. We showed in the present study that pioglitazone, one of TZDs attenuates ER stress and apoptosis in macrophage induced by palmitate, the most abundant SFA in the serum through up-regulation of SCD-1.These results suggest that reduction of SFA level may contribute anti-atherosclerotic effects of pioglitazone.

## Materials and Methods

### Materials

Dulbecco's Modified Eagle Medium (DMEM) was purchased from GIBCO-BRL, Invitrogen Co. (Carlsbad, CA, USA). Fetal bovine serum (FBS) was purchased from SAFC Biosciences Inc. (Lenexa, KS, USA.). Bovine serum albumin (BSA), palmitate, palmitoleate, pioglitazone, GW9662, and antibodies against PERK and α-Tubulin were purchased from Sigma-Aldrich Co. (St. Louis, MO, USA). Rosiglitazone was purchased from Wako Pure Chemical Industries, Ltd. (Osaka, Japan).

4-(2-Chlorophenoxy)-N-(3-(3-methylcarbamoyl)phenyl)piperidine-1-carboxamide, a SCD-1 inhibitor was purchased from BioVision (Mountain View, CA, USA). CAY10566 was purchased from Cayman.

An antibody against CHOP/GADD153 was purchased from Santa Cruz Biotechnology Inc. (Santa Cruz, CA, USA.). Antibodies against phosphorylated-PERK (pPERK), phosphorylated JNK (pJNK), JNK, cleaved caspase-3 were purchased from Cell Signaling Technology, Inc. (Danvers, MA, USA). Horseradish peroxidase-conjugated secondary antibodies were purchased from Vector Laboratories, Inc. (Burlingame, CA, USA). Palmitate and palmitoleate were solubilized in ethanol, and mixed with BSA at a 10:1 molar ratio and incubated in DMEM at 55°C for 3hr for conjugation. The control condition contained the same amount of BSA and ethanol.

### Preparation of peritoneal macrophages (PMs)

All procedures were approved by Animal Care and Use Committee, Kyushu University and conducted in accordance with the institutional guidelines. Mice were intraperitoneally injected with 2.0 ml of 3% thioglycollate. Four days later, the peritoneal cavity was lavaged with 6 ml PBS to retrieve infiltrated cells under anesthesia with injection of ketamine (100 mg/Kg) and xylazine (10 mg/Kg). After the lavage fluid was recovered and centrifuged (1000rpm, 5 min, 4°C), the pelleted cells were resuspended in DMEM containing 10% FBS. PMs were seeded in cell culture dish and incubated at 37°C, with 5% CO_2_ overnight. Non-adherent cells were removed by washing with PBS and adherent cells were used as PM.

### Cell culture

RAW264.7 cell [10], a murine macrophage cell line, was cultured in DMEM supplemented with 10% FBS, penicillin (100 units/ml) and streptomycin (100 μg/ml) containing 25mM glucose under the humidified condition of 95% air and 5% CO_2_ at 37°C.

### Western blot analysis

RAW264.7 cells were lysed in a lysis buffer containing RIPA (100mmol/L NaCl, 60 mmol/L Na_2_HPO_4_, 100 mmol/L NaF, 10 mmol/L EDTA, and 20 mmol/L Tris-HCl), 1% aprotinin, 0.5% pepstatin A, 1 mmol/L PMSF, and 0.05% leupeptin. Protein concentrations were determined with the bicinchoninic acid protein assay kit (Thermo Scientific, Rockford, IL, USA.). Cell lysates were heated in a sample buffer (62.5mmol/L Tris-HCl [pH 6.8], 10% glycerol, 2% SDS, 0.05% bromophenolblue, and 715 mmol/L 2-mercaptoethanol) at 95°C for 3 minutes, electrophoresed on 7 or 15% SDS-polyacrylamide gel, and transferred to polyvinylidene difluoride membrane (Immobilon-P, Millipore Corp, Billerica, MA, USA). The blots were blocked with TBS-T (20 mmol/L Tris-HCl [pH 7.6], 137 mmol/L NaCl, 0.1% Tween 20) containing 5% skim milk at room temperature for 30 minutes. Blots were detected by chemiluminescence system using ECL Western Blotting Detection Reagent (GE Healthcare, Chalfont St Giles, UK). The protein expression level was quantified by NIH ImageJ software. Activation of PERK and JNK was expressed as a ratio of pPERK to PERK and pJNK to JNK (which recognizes both phosphorylated and nonphosphorylated forms), respectively.

### Quantitative real-time reverse transcription polymerase chain reaction

Reverse transcription of RNA was performed with ReverTraAce (TOYOBO Co., Ltd., Osaka, Japan). Quantitative real-time reverse transcription polymerase chain reaction (qRT-PCR) was performed using THUNDERBIRD SYBR qPCR mix (TOYOBO) and the ABI PRISM 7500 Sequence Detection System (Applied Biosystems, Foster City, CA, USA). Relative expression levels were determined by standard curve method. Expression of *SCD-1* and *LXRα* was presented as the relative mRNA level to that of 18S rRNA. The sequences of PCR primers used in this study are as follow.

**Table 1:** 

*SCD-1*,	Forward 5’-TCTTGTCCCTATAGCCCAATCCAG-3’,
	Reverse 5’-AGCTCAGAGCGCGTGTTCAA-3’.
*LXRα*,	Forward 5’-TGGAGACGTCACGGAGGTACA-3’,
	Reverse 5’-CAGCTCATTCATGGCTCTGGA-3’.
ABCA1	Forward 5’- CCAGGAGCGTGTGAGCAAAG-3’
	Reverse 5’- TAATGACCAGTGTAGCAGGGACCA-3’
ABCG1	Forward 5’- GCTCCATCGTCTGTACCATCCA-3’
	Reverse 5’- CCTCAGATACGGCACGAGATTG-3’
18S rRNA,	Forward 5’-ACTCAACACGGGAAACCTCA-3’,
	Reverse 5’-AACCAGACAAATCGCTCCAC-3’

### Detection of Apoptosis

Apoptosis analysis was performed by using Annexin V-FITC Apoptosis Detection Kit I (BD Biosciences Pharmingen, San Diego, CA, USA) and BD FACS Calibur (Becton, Dickinson and Co., Franklin Lakes, NJ, USA) according to the manufacturer’s instructions. Cells were stained with Annexin V–FITC and propidium iodine (PI) for 15 minutes in the dark. Fluorescence of FITC or PI of 10000 cells was measured. Cells were counted and analyzed on BD Cell Quest PRO software (BD Biosciences).

### Statistical analysis

Statistical analysis was performed with one-way ANOVA and Fisher’s test, if appropriate. Data are shown as mean±S.E.M. Values of P < 0.05 were considered to be statistically significant.

## Results

### Palmitate induced ER stress and activated apoptotic signalling pathways

**Fig 1:**
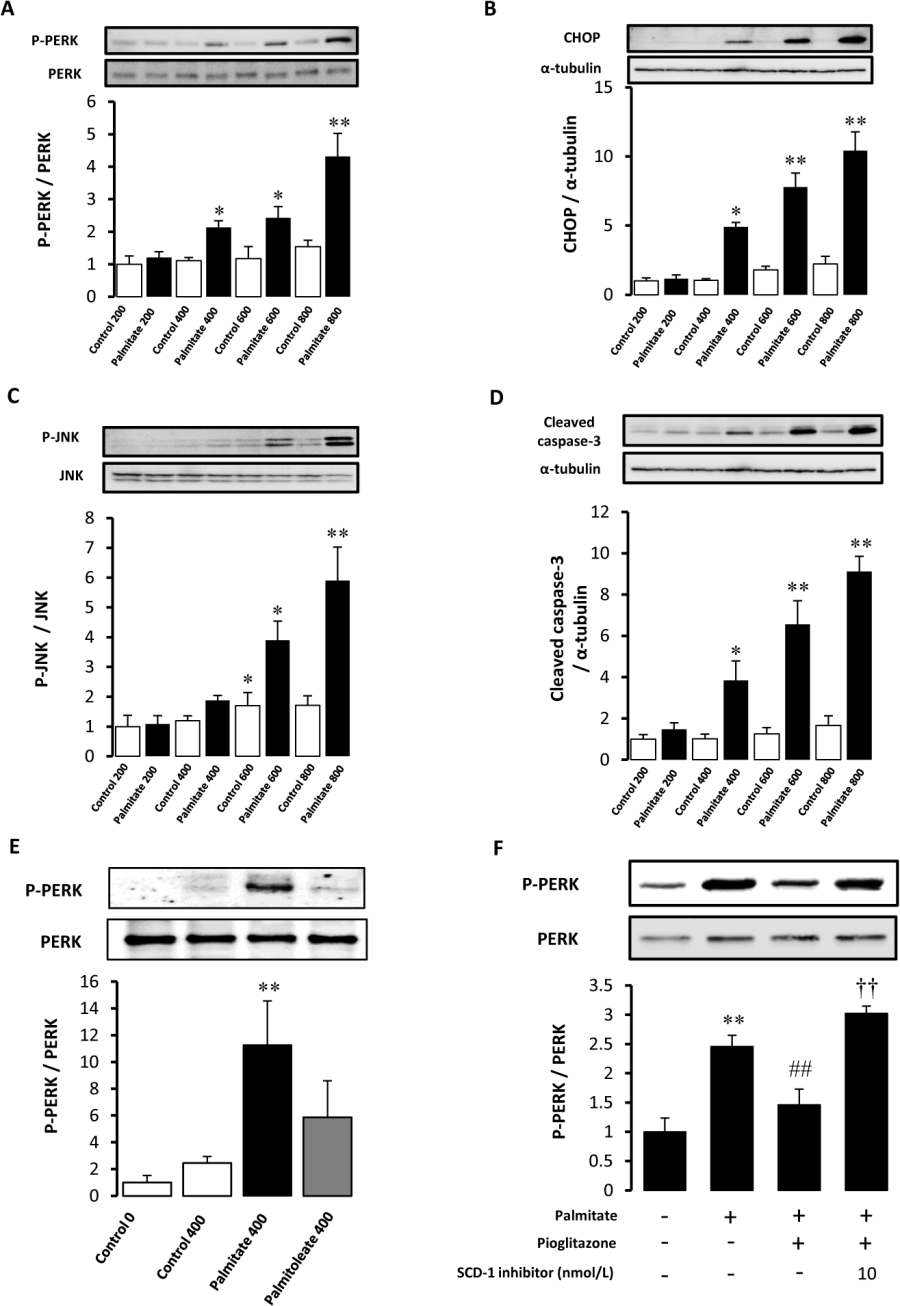
Effects of palmitate on ER stress and apoptotic signaling pathways. RAW264.7 cells were treated with various concentrations (μmol/L) of palmitate (Black bar) or corresponding amount of BSA (White bar) for 16 hours. Western blot analyses for Phospho (P)-PERK (A), CHOP (B), Phospho (P)-JNK (C), cleaved caspase-3 (D) were performed. (E) The effect of palmitoleate on PERK phosphorylation was compared with that of palmitate in RAW264.7 cells. The bar graphs indicate the ratio of P-PERK to PERK, CHOP to α-tubulin, P-JNK to JNK, and cleaved caspase-3 to α-tubulin, respectively. *P<0.05, ** P<0.01 vs control. n = 4. (F) The effect of palmitate on PERK phosphorylation was examined in peritoneal macrophages. The effects of pioglitazone and SCD-1 inhibitor were also examined. **p<0.01 vs control (palmitate (-), pioglitazone (-)), ##P<0.01 vs palmitate, ††P<0.01 vs Palmitate+Pioglitazone. n = 4.

To determine whether SFA induces ER stress in macrophage, we examined the effect of palmitate, the most abundant SFA in the serum, on phosphorylation of PERK in RAW264.7cells. Treatment with palmitate induced PERK phosphorylation in a dose-dependent manner (Fig 1A). Upon exposure to severe or prolonged ER stress, the PERK and IRE1 signalling pathways induce transcription and translation of proapoptotic factors such as CHOP and activate JNK pathway. This also results in caspase activation. As shown in Fig 1B–1D, palmitate induced CHOP expression, JNK phosphorylation and cleavage of caspase-3 in a dose-dependent manner. To confirm that the ER stress is specifically induced by saturated fatty acid, we examined the effect of palmitoleate, a mono-unsaturated fatty acid, on ER stress (Fig 1E). Palmitoleate failed to induce PERK phosphorylation, suggesting that saturated fatty acid may specifically induce ER stress.

We also confirmed that palmitate induced PERK phosphorylation in peritoneal macrophages (Fig 1F).

### Thiazolidinediones attenuated palmitate-induced ER stress and apoptotic signalling pathways

**Fig 2:**
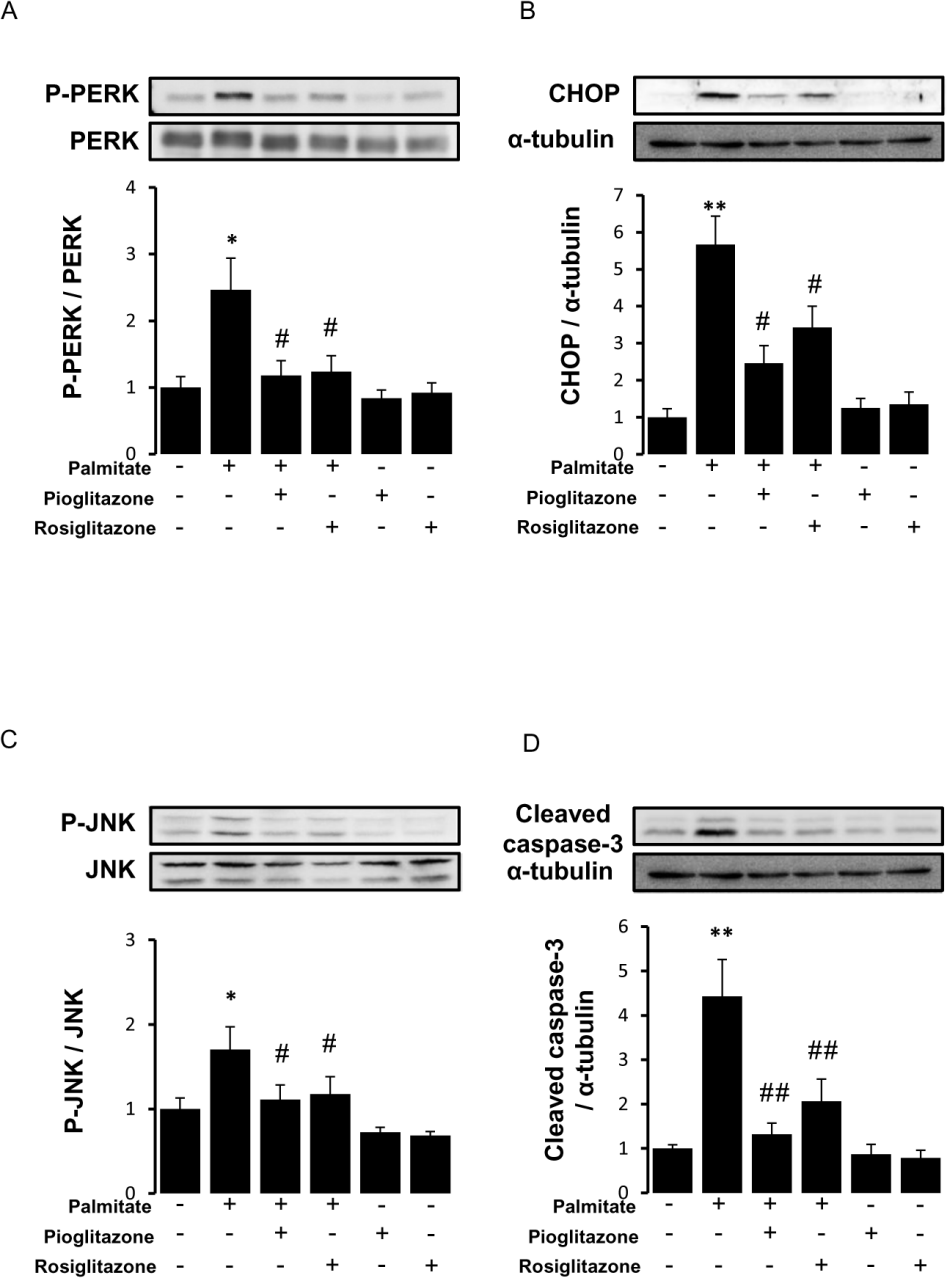
Effects of thiazolidinediones on palmitate-induced ER stress and apoptotic signaling pathways. RAW264.7 cells were pretreated with pioglitazone (10 μmol/L) or rosiglitazone (10 μmol/L) for 6 hours and stimulated with or without palmitate (400 μmol/L) for 16 hours.Western blot analyses for phospho (P)-PERK (A), CHOP (B), Phospho (P)-JNK (C) and Cleaved caspase-3 (D) were performed. The bar graphs indicates the ratio of P-PERK to PERK, CHOP to α-tubulin, P-JNK to JNK, and cleaved caspase-3 to α-tubulin, respectively. *P<0.05, ** P<0.01 vs control (palmitate (-), pioglitazone (-) and rosiglitazone (-)), #P<0.05, ## P<0.01 vs palmitate. n = 4.

Palmitate has been shown to induce ER stress and apoptosis in many cell types at concentrations between 250 μmol/L and 500 μmol/L [11, 12]. Because phosphorylation of PERK and JNK, expression of CHOP and cleavage of caspase-3 were significantly activated at 400 μmol/L of palmitate, we treated RAW264.7 cells with palmitate at a concentration of 400 μmol/L in the following experiments. The effects of vehicle (DMSO), pioglitazone (10 μmol/L) or rosiglitazone (10 μmol/L), another TZDs, on palmitate-induced ER stress and apoptotic signaling pathways were examined in RAW264.7 cells. Western blot analysis revealed that TZDs significantly inhibited palmitate-induced PERK phosphorylation, CHOP expression, JNK phosphorylation and cleavage of caspase-3 (Fig 2A–2D). It is suggested that anti-ER stress effects are mediated by PPARγ activation, because rosiglitazone showed the same anti-ER stress effects as pioglitazone. We, therefore, focused on pioglitazone in the following experiments.

### A PPARγ antagonist reversed anti-ER stress effects of pioglitazone

**Fig 3:**
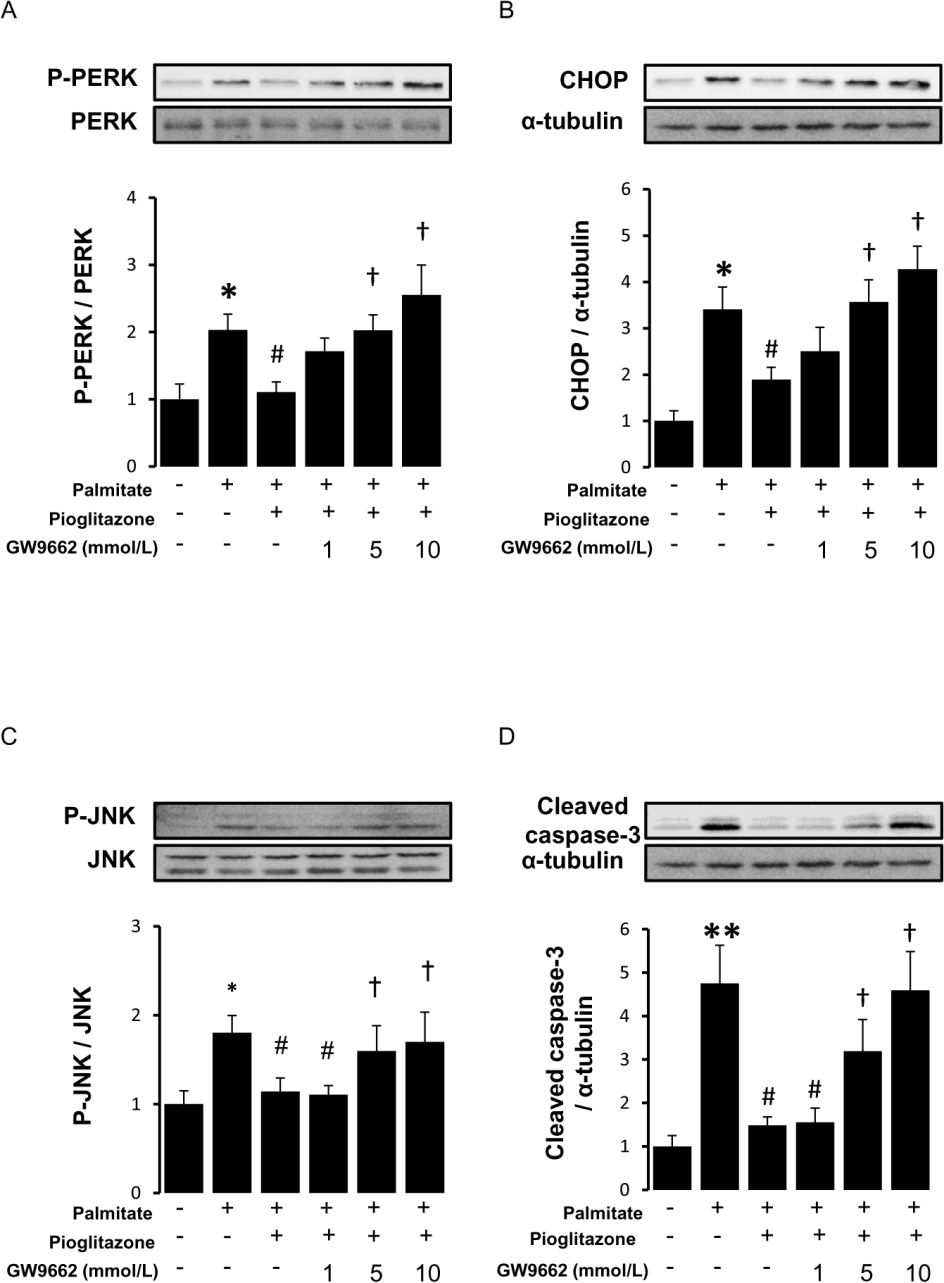
Reversion of the anti-ER stress and anti-apoptotic effects of pioglitazone by GW9662. RAW264.7 cells were pretreated with GW9662 (1, 5, 10 μmol/L) for 1 hour and incubated with pioglitazone (10 μmol/L) for 6 hours. Then, cells were stimulated with palmitate (400 μmol/L) for 16 hours. Western blot analyses for phospho (P)-PERK (A), CHOP (B), phospho (P)-JNK (C), cleaved caspase-3 (D) were performed. The bar graphs indicate the ratio of P-PERK to PERK, CHOP to α-tubulin, P-JNK to JNK, and cleaved caspase-3 to α-tubulin, respectively. *P<0.05, ** P<0.01 vs control (palmitate (-), Pioglitazone (-) and GW9662 (-)), #P<0.05 vs palmitate, †P<0.05, ††P<0.01 vs Palmitate+Pioglitazone. n = 4.

We next examined the effect of GW9662, a selective PPARγ antagonist. The anti-ER stress and anti-apoptotic effects of pioglitazone were blocked by GW9662 in a dose-dependent manner (Fig 3A–3D), suggesting that the effect of pioglitazone was mediated by PPARγ activation.

### Pioglitazone increased SCD-1 mRNA level in macrophage

**Fig 4:**
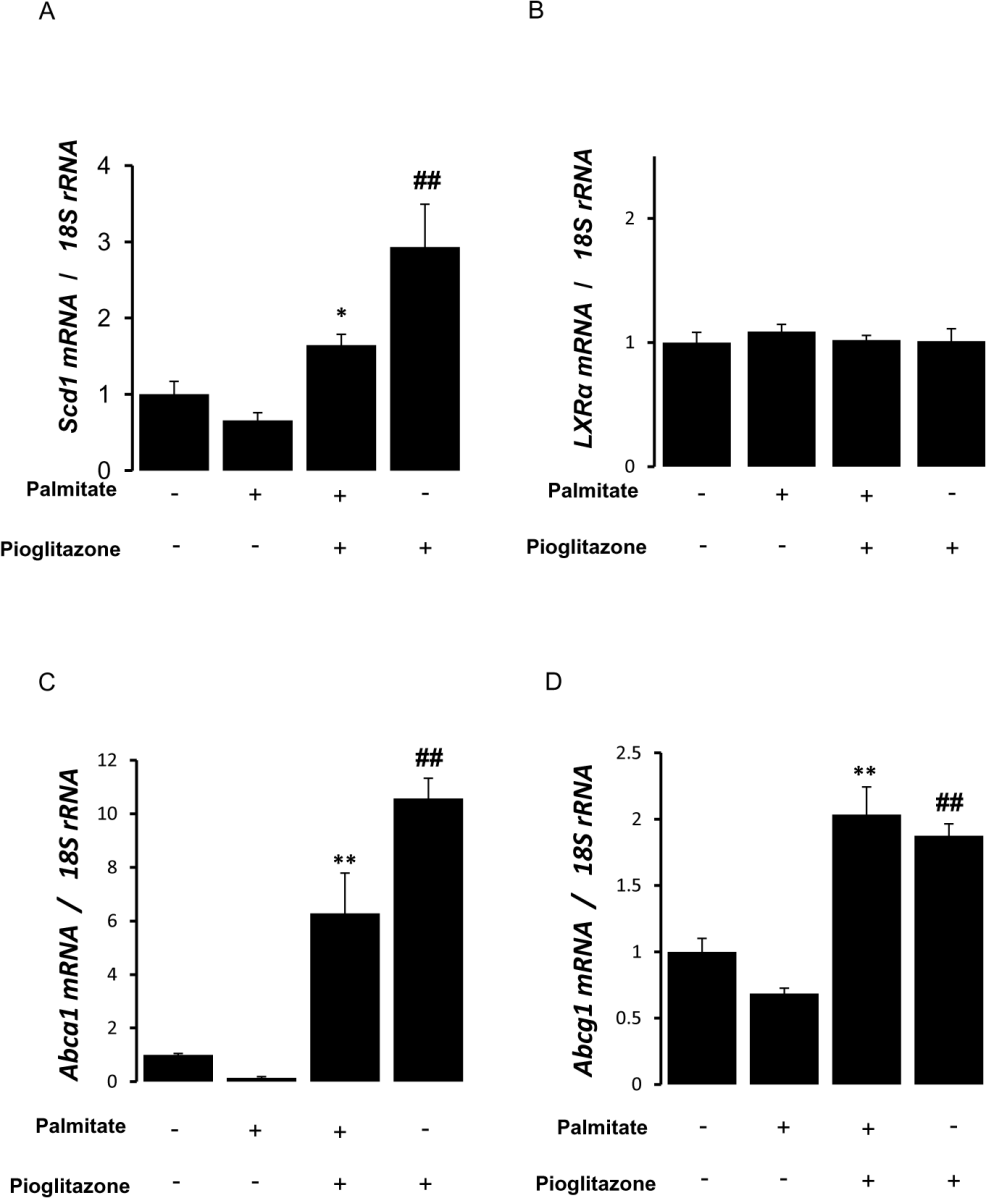
Effects of pioglitazone treatment on SCD-1 and LXRα mRNA expression. RAW264.7 cells were pretreated with pioglitazone (10 μmol/L) for 6 hours and stimulated with or without palmitate (400 μmol/L) for 16 hours. Expression of SCD-1 (A), LXRα, Abca1 (C) and Abcg1 (D) mRNA was determined by qRT-PCR. Expression of SCD-1, LXRα, Abca1, and Abcg1 mRNA was presented as the relative level to that of 18S rRNA. **p<0.01, *p<0.05 vs palmitate, ##p<0.01 vs control (palmitate (-) and pioglitazone (-)). n = 4.

We investigated whether pioglitazone modulates expression of SCD-1 in RAW264.7 cells. Palmitate slightly decreased SCD-1 mRNA level. Pretreatment with pioglitazone resulted in a significant increase in SCD-1 mRNA level in the presence or absence of palmitate (Fig 4A). There was no change in LXRα mRNA level after treatment with palmitate and/or pioglitazone in RAW264.7 cells (Fig 4B). However, the expression of ABCA1 and ABCG1, target genes of LXRα, was up-regulated by pioglitazone treatment (Fig 4C and 4D). These data suggest that LXRα pathway is activated by pioglitazone.

### SCD-1 inhibition blocked anti-ER stress and anti-apoptotic effects of pioglitazone

**Fig 5:**
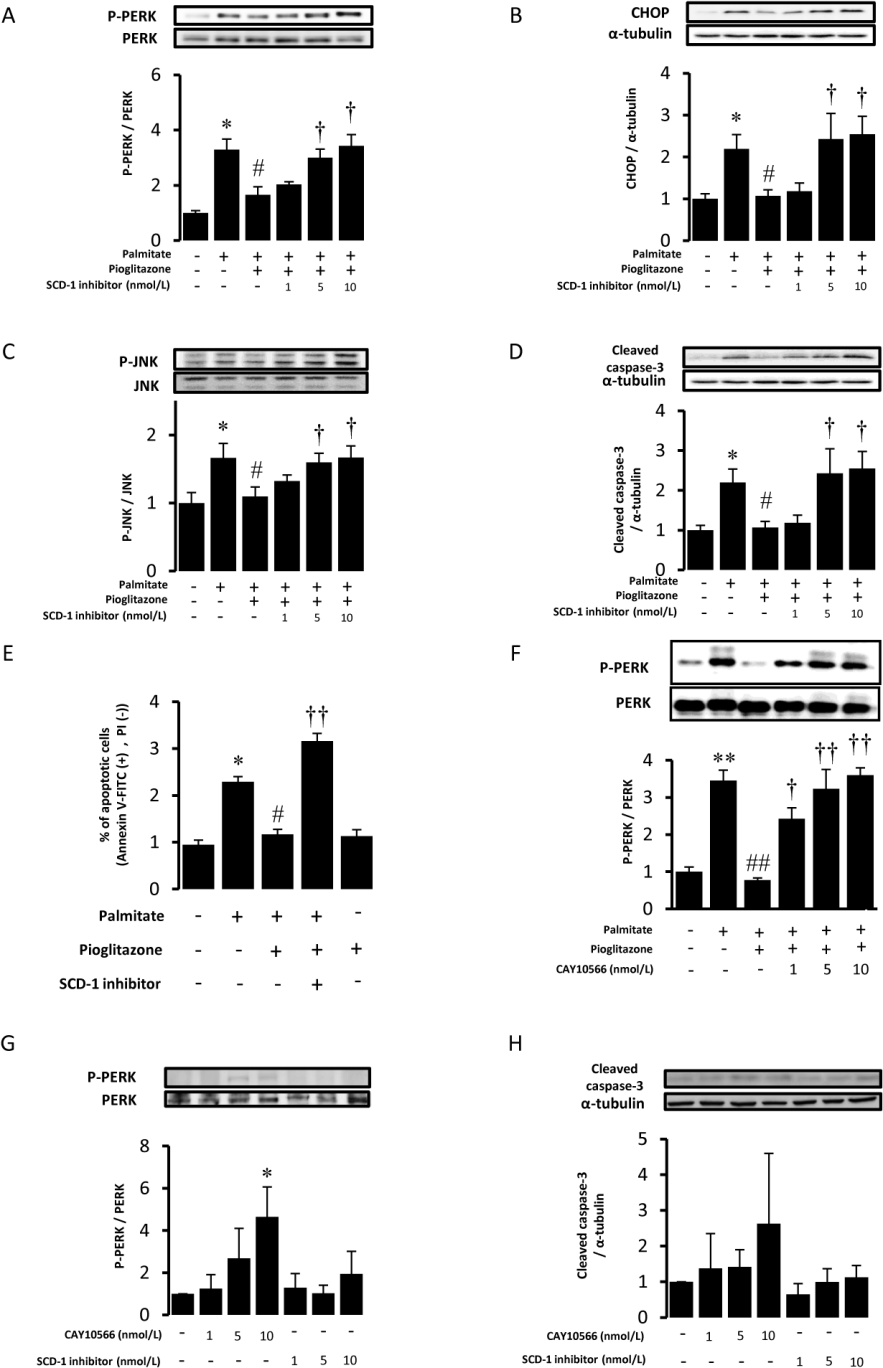
Reversion of the anti-ER stress and anti-apoptotic effects of pioglitazone by a SCD-1 inhibitor. RAW264.7 cells were pretreated with a SCD-1 inhibitor (1, 5, 10 nmol/L) for 1 hour and incubated with pioglitazone (10 μmol/L) for additional 6 hours. Then, cells were stimulated with palmitate (400 μmol/L) for 16 hours. Western blot analyses for phospho (P)-PERK (A), CHOP (B), Phospho(P)-JNK (C), cleaved caspase-3 (D) were performed. The bar graphs indicate the ratio of P-PERK to PERK, CHOP to α-tubulin, P-JNK to JNK, and cleaved caspase-3 to α-tubulin, respectively. *P<0.05 vs control (palmitate (-), pioglitazone (-) and SCD-1inhibitor (-)). #P<0.05 vs palmitate. †P<0.05 vs Palmitate+Pioglitazone. n = 4. (E) RAW264.7 cells were pretreated with pioglitazone (10 μmol/L) for 6 hours and stimulated with or without palmitate (400 μmol/L) for 16 hours. SCD-1 inhibitor was added 1 hour before pioglitazone treatment. The number of cells stained positively with Annexin V–FITC and negatively with PI was counted by flowcytometry. * P<0.05 vs control (palmitate (-), pioglitazone (-) and SCD-1inhibitor (-)), ##P<0.01 vs palmitate, ††P<0.01 vs Palmitate+Pioglitazone. n = 3. (F) RAW264.7 cells were pretreated with CAY10566, another SCD-1 inhibitor (1, 5, 10 nmol/L) for 1 hour and incubated with pioglitazone (10 μmol/L) for additional 6 hours. Then, cells were stimulated with palmitate (400 μmol/L) for 16 hours. Western blot analyses for phosphor (P)-PERK and PERK were performed. The bar graphs indicate the ratio of P-PERK to PERK. **P<0.01 vs control (palmitate (-), pioglitazone (-) and SCD-1inhibitor (-)). ##P<0.01 vs palmitate. ††P<0.01, †P<0.05 vs Palmitate+Pioglitazone. n = 4. RAW264.7 cells were treated with a SCD-1 inhibitor or CAY10566 (1, 5, 10 nmol/L) for 23 hours. Western blot analyses for phospho (P)-PERK (G), cleaved caspase-3 (H) were performed. The bar graphs indicate the ratio of P-PERK to PERK and cleaved caspase-3 to α-tubulin, respectively. *P<0.05 vs control. n = 4.

Finally, to determine the role of SCD-1 in palmitate–induced ER stress and apoptosis, we assessed the effect of an inhibitor of SCD-1. Suppression of phosphorylation of PERK and JNK, CHOP expression, and cleavage of caspase-3 by pioglitazone was reversed by an SCD-1 inhibitor in a dose-dependent manner in RAW264.7 cells (Fig 5A–5D). The same result was obtained in peritoneal macrophages in terms of PERK phosphorylation (Fig 1F). We also examined whether pioglitazone attenuates palmitate-induced macrophage apoptosis. Annexin V specifically binds to phosphatidylserines that are exposed at the cell surface in the preapoptotic stage. Staining with PI indicates dead cells. The number of RAW264.7 cells stained positively with Annexin V–FITC and negatively with PI was increased by incubation with palmitate and significantly reduced by pretreatment with pioglitazone. Reduced apoptosis by pioglitazone was reversed by inhibitor of SCD-1(Fig 5E). These data suggest that anti-ER stress and anti-apoptotic effects of pioglitazone may be mediated by SCD-1 up-regulation. We examined the effect of CAY10566, another SCD-1 inhibitor [13], on pioglitazone suppression of PERK phosphorylation. CAY10566 also reversed PERK phosphorylation suppressed by pioglitazone, indicating that the amelioration of ER stress by pioglitazone was mediated by SCD-1 pathway (Fig 5F). We examined the effects of SCD-1 inhibitor and CAY10566 on PERK phosphorylation and cleavage of caspase 3 at the baseline. SCD-1 inhibitor did not show any effect on PERK phosphorylation and cleavage of caspase 3. However, CAY10566 slightly induced PERK phosphorylation and cleavage of caspase 3(Fig 5G and 5H).

## Discussion

Although the previous studies suggest that PPARγ agonists increase cholesterol efflux from macrophages and decrease cytokine expression, its involvement in ER stress has not been studied well. We demonstrated in the present study that PPARγ agonists attenuated palmitate-induced ER stress and apoptosis in macrophages. Furthermore, we showed that pioglitazone-induced marked resistance to ER stress and apoptosis is dependent on SCD-1. To our knowledge, we firstly reported that PPARγ agonists attenuated SFA-induced ER stress and apoptosis in macrophage.

Dietary SFAs have been implicated in promoting the metabolic syndrome and atherosclerotic cardiovascular diseases [14]. It has been reported that SFAs have lipotoxic effects leading to ER stress and apoptosis in many cell types [11, 12]. On the other hand, MUFAs appear less toxic and can reduce SFA-induced ER stress [11]. SCD-1 plays a key role in preventing lipotoxic effects of SFAs, as it converts lipotoxic SFAs to less toxic MUFAs. It has been reported that upregulation of SCD-1 prevents ER stress in endothelial cells [15], whereas knockdown of SCD-1 enhanced palmitate-induced ER stress in insulin secreting cells [16]. SCD-1 deficiency increased atherosclerotic lesion in low-density-lipoprotein receptor (LDLR) knockout mice with an increase in plasma ICAM-1 and IL-6 levels [17]. It was also reported that antisense oligonucleotide for SCD-1 accelerates atherogenesis in LDLR-deficient mice [18]. Based on these reports, it is suggested that the mechanism by which PPARγ agonists stabilize atherosclerotic plaque may partially result from reducing ER stress through up-regulation of SCD-1.

Although statistically insignificant, palmitate suppressed SCD1, ABCA1 and ABCG1 mRNA levels. We did not examine the signaling pathway of palmitate in this study. However, it is suggested that palmitate activates toll-like receptor (TLR) 4 [19]. And previous studies showed that lipopolysaccharide, another ligand for TLR4, suppressed expression of ABCA1 [20] and ABCG1 [21] in macrophages. Therefore, it is possible that down-regulation of these genes may be due to activation of TLR4 by palmitate.

However, the effect of PPARγ agonists on ER stress seems to be controversial. Pioglitazone improved pancreatic islet function such as insulin release in diabetic mice through reduction of ER stress [22]. Pioglitazone also reduced ER stress in the liver of diabetic mice [23]. Whereas 15-deoxy-Δ^12,14^-prostaglandin J_2_, another PPARγ agonist, activated expression of unfolded protein response-responsive genes [24]. TZD18, a PPARγ/δ dual ligand, activated ER stress response and induced growth arrest and apoptosis in breast cancer cells [25]. The mechanism for the differential response to PPAR agonist is not clear. Diabetic mice were treated with pioglitazone and ER stress was examined in vivo in the former 2 studies, in which showed that PPARγ reduced ER stress [22, 23]. In contrast, other 2 studies showing that PPARγ induced ER stress [24, 25] examined the effects of PPARγ agonists on cells derived from tumors such as RINm5F cell line derived from rat insulinoma and breast cancer cell line, MCF-7. Therefore, it may be possible to assume that the effect of PPARγ agonists on ER stress may be dependent on the stimuli that induce ER stress and cell or tissue type. Alternatively, different PPARγ agonist may show differential effects on ER stress, although we showed that pioglitazone and rosiglitazone have the same effects on palmitate-induced ER stress so far as we examined.

It was reported that the *Fabp4*-deficient macrophages showed a marked increase in SCD-1 and LXR activity [8]. SCD-1 is known to be one of the target molecules of LXR [26]. Although pioglitazone did not increase LXRα mRNA levels, expression of target genes of LXRα such as ABCA1 and ABCG1 was increased, indicating that LXR pathway is activated. Therefore, we could not exclude the role of LXR in the pioglitazone-induced upregulation of SCD-1 at this point. It is reasonable, however, to assume that both LXR and PPARγ contribute to pioglitazone-induced up-regulation of SCD1 gene, because PPARγ response element is present in the SCD1 gene promoter [24] and a LXR agonist such as TO-901317 was reported to induce SCD1 expression [26].

Although a previous report showed that a putative PPAR response element is located in the SCD-1 gene promoter [27], the effect of TZDs on SCD-1 expression is controversial. It was reported that TZDs suppress SCD-1 expression in 3T3-L1 adipocytes [28]. Hepatic SCD-1 expression was not affected by pioglitazone in obese Zucher fa/fa rats [29]. However, SCD-1 mRNA expression in adipose tissue from subjects with type 2 diabetes was increased after treatment with rosiglitazone [30]. It is difficult to reconcile these contradicting results, but it is possible that the effect of TZDs on SCD-1 expression may be species- or tissue-dependent as the effect of TZDs on ER stress is not consistent.

Two inhibitors of SCD1 showed differential effects on basal PERK phosphorylation and Caspase3 cleavage (Fig 5G and 5H). A previous report showed that CAY10566 induced phosphorylation of ATF4, the transcription factor downstream of the PERK pathway, and production of CHOP in VSMC [13]. Although the reason for this differential effect is not immediately clear, it was suggested that basal SCD-1 activity may play some role in the reduction of ER stress.

Macrophage death plays Janus-faced roles in atherogenesis. Several studies have shown that macrophage apoptosis in early atherosclerotic lesions is associated with smaller lesion size and less plaque progression [31]. This phenomenon is explained by an efficient removal of apoptotic cells by neighboring macrophages. In contrast, macrophage death is reported to promote plaque necrosis in advanced atherosclerotic lesions, in which ER stress of macrophage is believed to play a critical role. For example, macrophage apoptosis and plaque necrosis were reduced in CHOP-deficient apolipoprotein E knockout mice [32].

A previous study showed that pioglitazone reduced cardiovascular events in patients with diabetes [33]. The result is consistent with the idea that macrophage apoptosis induces plaque vulnerability and pioglitazone reduces macrophage apoptosis through attenuation of ER stress, which was shown in this study. However, it has not been determined whether pioglitazone up-regulates macrophage SCD-1 and suppresses apoptosis in vivo. Further studies are needed.

In conclusion, we showed in the present study that PPARγ agonists attenuated palmitate-induced macrophage ER stress and apoptosis. These effects depend on SCD-1 that may modulate fatty acid profile in macrophage, and partially contribute to the plaque stability in atherosclerotic lesion.
